# Exogenous Cx43 expression decrease cell proliferation rate in rat hepatocarcinoma cells independently of functional gap junction

**DOI:** 10.1186/1475-2867-9-22

**Published:** 2009-08-13

**Authors:** Marisa Ionta, Raphael Adolpho Sant'ana Ferreira, Sandra Cristina Pfister, Gláucia Maria Machado-Santelli

**Affiliations:** 1 Department of Cell and Developmental Biology, Institute of Biomedical Science University of Sao Paulo, Brazil; 2 Av. Prof. Lineu Prestes, 1524, ICB I, Sao Paulo, SP – Brazil – 05508-901

## Abstract

**Background:**

Gap junction intercellular communication (GJIC) is considered to play a role in the regulation of homeostasis because it regulates important processes, such as cell proliferation and cell differentiation. A reduced or lost GJIC capacity has been observed in solid tumors and studies have demonstrated that GJIC restoration in tumor cells contribute to reversion of the transformed phenotype. This observation supports the idea that restoration of the functional channel is essential in this process. However, in the last years, reports have proposed that just the increase in the expression of specific connexins can contribute to reversion of the malign phenotype in some tumor cells. In the present work, we studied the effects of exogenous Connexin 43 (Cx43) expression on the proliferative behavior and phenotype of rat hepatocarcinoma cells.

**Results:**

The exogenous Cx43 did not increase GJIC capacity of transfected cells, but it was critical to decrease the cell proliferation rate as well as reorganization of the actin filaments and cell flattening. We also observed more adhesion capacity to substrate after Cx43 transfection.

**Conclusion:**

Cx43 expression leads to a decrease of the growth of the rat hepatocellular carcinoma cells and it contributes to the reversion of the transformed phenotype. These effects were independent of the GJIC and were probably associated with the phosphorylation pattern changes and redistribution of the Cx43 protein.

## Background

The survival of multicellular organisms depends on the tissue homeostasis. The gap junction intercellular communication (GJIC) has long been proposed to play an important role in the control of cell proliferation, differentiation and apoptosis. Among the mechanisms mediating cell-to-cell interactions, GJIC is unique in the sense that cells directly transfer small molecules (<1000Da) from the inside of one cell to that of neighboring cells. A gap junction channel consists of two juxtaposed hemichannels provided by each adjacent cell. The connexon is composed of six subunit proteins called connexins (Cx) [1,2] which are coded by a multigene family. In humans, at least 21 members have been described [3] and their expression is tissue-specific [2,4].

In the liver, three connexins are expressed depending on the cell type and its position in the lobule. Both Cx32 and Cx26 are expressed by hepatocytes; Cx32 is expressed by hepatocytes in the hepatic acinus, while Cx26 is expressed by hepatocytes localized in the periportal spaces. Cx43 is normally expressed by oval cells, endothelial cells and biliary cells, but not hepatocytes. However, when the hepatocytes are cultured "in vitro", they can express Cx43 instead of Cx26 and Cx32. For example, BRL and REL cell lines are derived from normal liver and express Cx43 as the major gap junction protein [5,6].

Deficient GJIC is frequently observed in tumor cell lines [7-9] and considering that GJIC plays a key role in the homeostasis of multicellular organisms, it is not surprising that disruption of gap junction communication is involved in the carcinogenesis process [10].

Tumor cells (rat glioma, human lung giant carcinoma and human rhabdomyosarcoma) transfected with connexin 43 cDNA recovered GJIC capacity and they showed cell growth inhibition [11-13]. In the liver, these approaches have been less explored. At the moment, it is not clear whether the exogenous expression of Cxs would restore the gap junction and/or contribute to cell growth inhibition. Eghbali et al.[14] did not observe growth inhibition in Cx32 transfected cells, however when these cells were injected in animals they induced smaller tumors than the non-transfected cells. This discrepancy can be related to Cxs expression differential pattern observed "in vitro" and "in vivo" (Cx43 and Cx32, respectively). Considering that studies "in vitro" still represent an important tool in the liver cancer investigations and that Cx43 represents the major form expressed in normal liver cell lines and hepatoma cells, it would be interesting to further explore the relationship among the exogenous expression of Cx43, GJIC and proliferative behavior. The present work evaluated the effects of exogenous Cx43 expression on the proliferation of rat hepatocarcinoma cells as well as its influence on the cellular phenotype.

## Materials and methods

### Cell culture, growth curve and BrdU incorporation

The cells lines used were HTC (derived from rat hepatocarcinoma) and BRL3A (derived from normal rat liver). The cultures were maintained in DMEM (Dulbecco^'^s Modified Eagle's Minimum Essential Medium, Sigma, CA, USA), supplemented with 5% fetal bovine serum (FBS, Cultilab, Sao Paulo, Brazil). The cells were grown in a 37°C humidified incubator containing 5% CO_2_. Subcultures were performed every 2 to 3 days. For growth curve and BrdU incorporation the cells were plated in 35 mm Petri dishes at 5 × 10^3^cells/plate. The cells were counted on the 2^nd^, 4^th^, 7^th ^and 10^th ^days in culture. The BrdU (100 μM) (Sigma) was incorporated for 1 hour in the 5^th ^day in culture. The cells were fixed with ethanol: acetic acid (3:1) for 30 minutes and the immunofluorescence assays were performed.

### Construction of Cx43-wt cDNA and Transfection conditions

The coding sequence of *connexin 43 *(Genbank accession number NM012567) was amplified by polymerase chain reaction [15] from the clone G2A (kindly provided by Dr David Paul, Harvard Medical School). The forward primer: 5'-CGC GGA TCC ATG GGT GAC TGG AGT GCC TTG-3' and a single reverse primer: 5'-CCG GAA TTC TTA ATT CTC CAG GTC ATC AGG-3' were used. The complete open reading frame of connexin 43 cDNA, with the stop codon, was fused to pcDNA3 vector (Invitrogen, Carlsbad, CA, USA). The recombinant plasmid was verified by DNA sequencing on ABI-PRISM 377 (Applied Biosystems).

The HTC cells were seeded at 5 × 10^4 ^in 35 mm dishes. Cultures with 60–70% confluence were transfected with Lipofectin reagent according to the manufacture's specifications (Invitrogen-Life Technologies).

### Immunofluorescence

The cells were cultured on coverslips into 35 mm dishes at 5 × 10^3^cells/plate. On the 5^th ^day, the DMEM was removed and the cells were washed with phosphate-buffered saline (PBSA), and fixed with 3.7% formaldehyde for 30 minutes and processed to labeling of interest. For Cx43 immunolabelling the cells were treated with cold acetone for 5 minutes and were once again washed in PBSA. Mouse anti-connexin 43 (Sigma) was incubated overnight in a wet chamber at 4°C. The cells were washed with PBSA and incubated with secondary antibody (anti-mouse IgG-FITC, Sigma) for 2 hours. The cells were once again washed with PBSA and the coverslips were mounted on histological slides with Propidium Iodide (10 μg/mL) and Vecta-Shield (Vector Laboratories, Burlingame, CA, USA). The actin filaments were labeled with TRITC-phalloidin for 20 minutes, after cell membranes had been treated with Triton X-100 (0.5%) for 10 minutes. The analyses were performed using a confocal laser scanning microscope (LSM 510, Zeiss, Germany). The lasers used were argon (488 nm) and helium-neon (543 nm) connected to an inverted fluorescence microscope (Axiovert 100 M, Zeiss).

### Scrape Loading and Dye Transfer Assay

Intercellular coupling was determined by a scrape loading and dye transfer (SL/DT) technique as described by El-Fouly et al. [16] with some modification. The cells were cultured in 35 mm Petri dishes. The medium was removed from the dishes and the cell layer was washed with PBSA. Lucifer Yellow solution (10 mg/mL) (Sigma) was added to the dishes and the cells were scraped with a surgical blade. After 5 minutes, the dye was removed, the dishes were once again washed with PBSA and the cells were fixed with 3.7% formaldehyde in PBSA. The cells were examined in a Nikon fluorescence microscope. The quantification of intercellular communication was accomplished by counting the number of fluorescent cells [17].

### Immunoblot

The cells were cultured in 75 cm^2 ^flasks. On the 5^th ^day of culture, the medium was removed, the cells were washed with cold PBSA and scraped in 10 mM Sodium Bicarbonate buffer supplemented with protease inhibitors (Sigma). Then 40 mM NaOH was added and after 30 minutes the cell lysate was sonicated (2 seconds at 20%) and then it was centrifuged (20.000 g) at 4°C for 20 minutes. The pellet was washed with sodium bicarbonate buffer and resuspended in sample buffer (2×). BCA assay (Pierce Biotechnology, Rockford, USA) was used to determinate the total protein concentration. Thirty micrograms (30 μg) of protein were separated by SDS-PAGE (10%) and transferred to PVDF membrane (Amersham Bioscience) (100 V, 250 mA, for 2 hours). The blocking solution (TBS, 5% milk and 0.05% Tween 20) was added for 1 hour at 4°C. The membrane was incubated with anti-connexin 43 monoclonal antibody (Sigma) diluted at 1:500, at 4°C overnight. After washing with TTBS (3 × 5 minutes), it was incubated with anti-mouse peroxidase conjugated for 2 hours at 4°C. The immunoreactive bands were visualized with the ECL Western blotting detection Kit (Amersham Pharmacia) according to the manufacture's instructions. Alpha-tubulin (α-tubulin) was used as a control to confirm equal loading conditions. Densytometric analyses were performed using an ImageJ program.

### Flow cytometry

The cells were cultured in 25 cm^2 ^flasks until the 5^th ^day. The cells were detached from the substrate using trypsin and centrifuged (5 minutes at 1000 rpm). The pellet was resuspended in PBSA and the cells were fixed with cold methanol (70%). After washes in PBSA, the cell suspension was stained with a dye solution (PBSA containing 3 mg/mL RNAase and 30 ug/mL propidium iodide) and the DNA was quantified by a flow cytometer (Becton Dickinson, Sao Jose, CA, USA) after 1 hour staining.

### Statistical Analysis

The quantitative data are presented as mean ± standard error. The ANOVA test was performed. In following Bonferroni's multiple comparisons test. The software used was Graphpad Prism (GraphPad Software, Inc., San Diego, CA, USA).

## Results

### Cell Morphology

**Figure 1:**
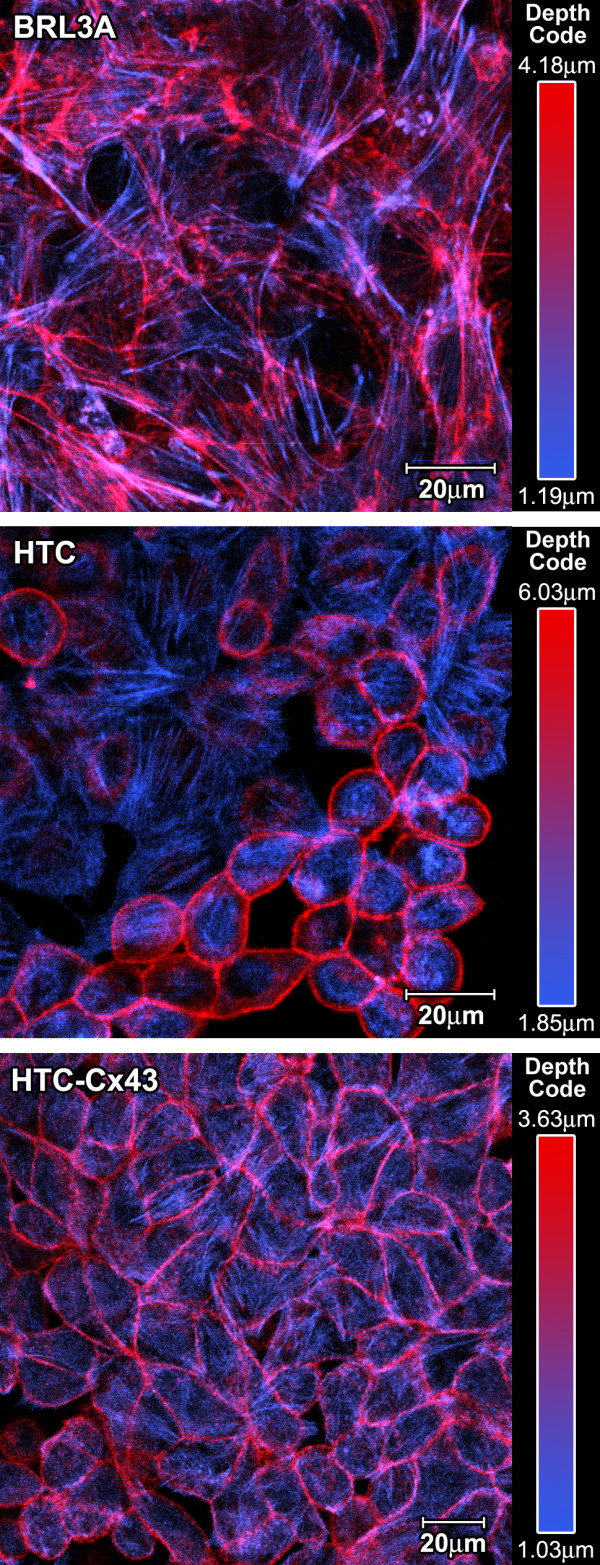
**Microfilament distribution**: BRL3A (A), HTC (B) and HTC-Cx43 (C) cells. In order to improve the observation of F-actin distribution pattern we merged one basal (blue) with one apical (red) optical slice, after artificial color attribution to each slice. (Their position in Z axis is indicated by μm).

HTC is a pleomorphic cell lineage, presenting cells with different morphological features. Some cells grow partially adhered to the substratum, forming rope-like structures while others grow more adhered to it and these were called type 1 and type 2 cells, respectively. These morphological differences are well evidenced when cytological preparations are stained with TRITC-phalloidin. Actin filaments in type 1 cells were concentrated near the plasma membrane while the actin filaments in type 2 cells were distributed all over the cytoplasm, including the cell cortex. Type 2 cells were the predominant cell type in the transfected cell line (HTC-Cx43) and they exhibit "stress fibers" in addition to the F-actin spread in the cytoplasm. It is important to point out that this is a characteristic of BRL3A cells (a normal liver cell line) but not of HTC cells. The microfilaments distribution pattern is quite evident in figure 1, which presents two different optical slices in Z axis merged in single images (basal in blue and apical in red) to show more clearly both cell types.

### Cell proliferation

**Figure 2:**
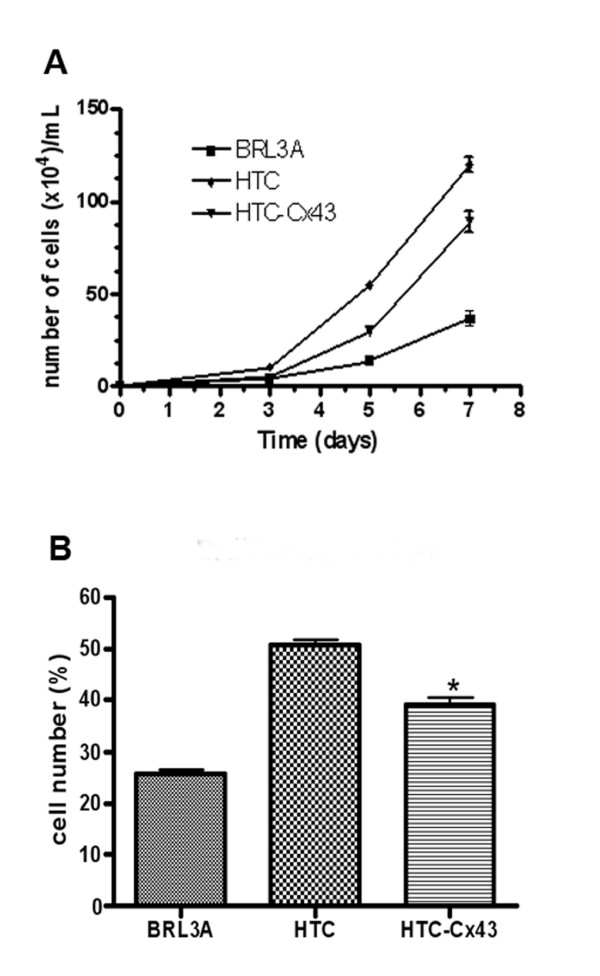
**Cell proliferation**: A) Growth curve of the BRL3A, HTC and HTC-Cx43 cell lines (mean ± SD of the triplicates data); B) Cell population in the S-phase determined by BrdU incorporation (mean ± SD for 3 independent experiments. The asterisk indicates a significant difference compared to the HTC with HTC-Cx43, * p < 0.05).

**Figure 3:**
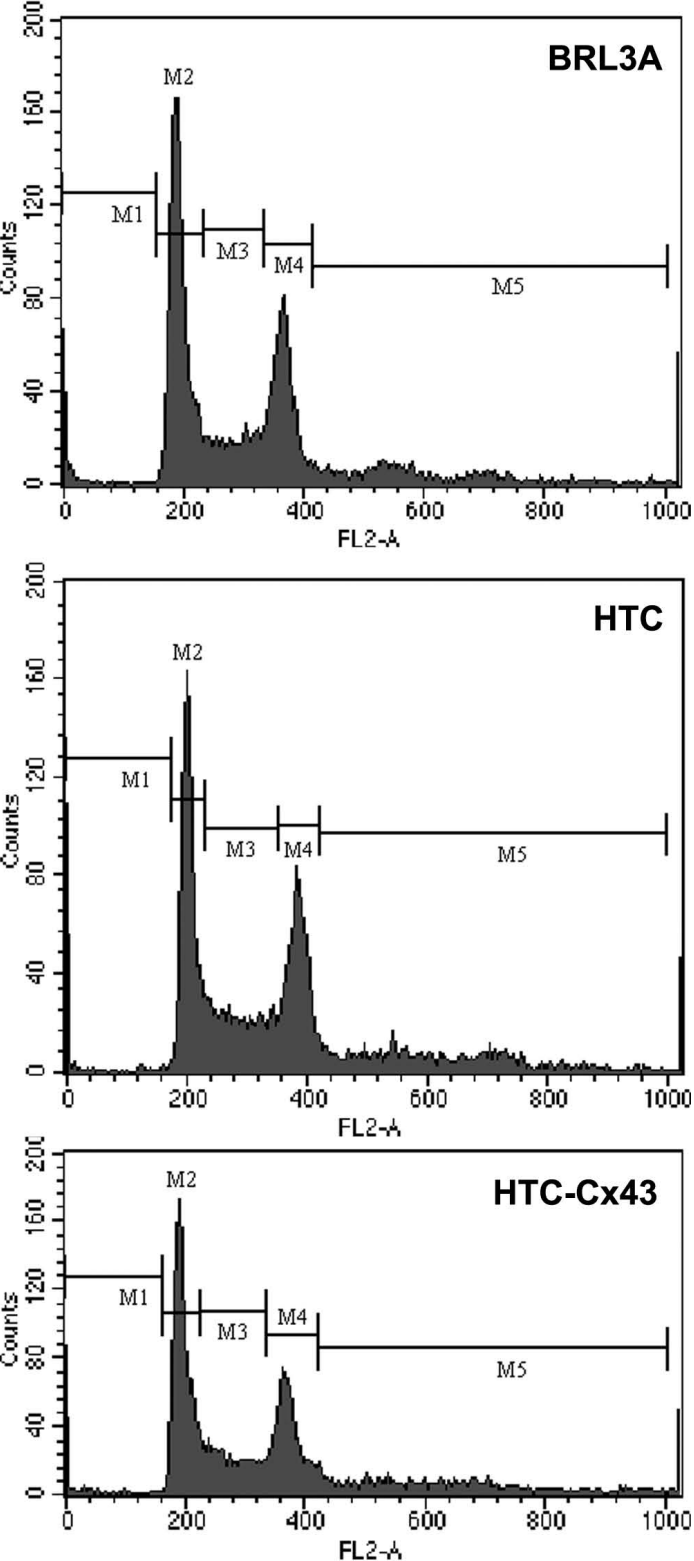
**DNA distribution histograms obtained from Flow cytometry**. The cell population in the s phase corresponding to 18%, 27.5% and 22% to BRL3A, HTC and HTC-Cx43 cell line, respectively. The data shown are representatives of three independent experiments.

The population doubling time, calculated for each cell line from its growth curve (Figure 2A), was approximately 10 h, 12 h and 20 h for HTC, HTC-Cx43 and BRL3A, respectively. The 5^th ^day was established as a representative point of exponential growth phase for further investigations of proliferation ability. The BrdU incorporation indexes in cytological preparations were 46%, 36% and 25% to HTC, HTC-Cx43 and BRL3A cells, respectively (Figure 2B). S-phase population evaluated by flow cytometry corresponded to 27.5%, 22% and 18% for HTC, HTC-Cx43 and BRL3A cell line, respectively (Figure 3). We observed, using different methods, that the proliferation rate was always lower in the transfected cells (HTC-Cx43) than in the hepatocarcinoma cells (HTC).

### Connexin 43 expression

**Figure 4:**
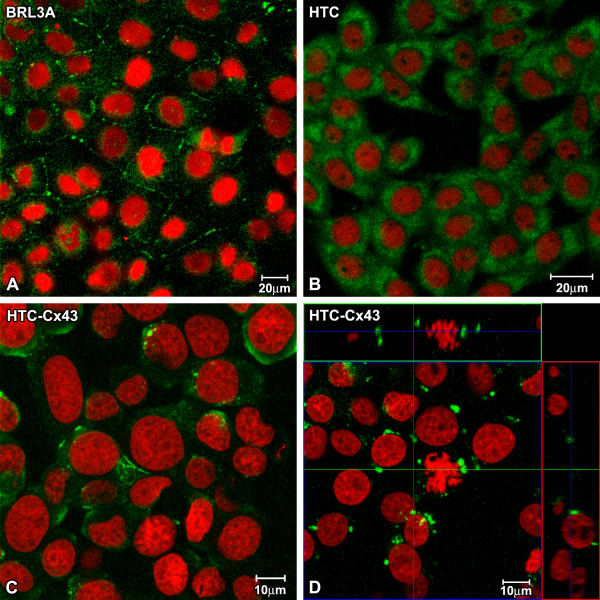
**Images obtained by Laser Scanning Confocal Microscopy showing Cx43 immunoreaction (green)**. The Cx43 is observed in cell surface in BRL3A (A) and HTC-Cx43 (C and D), while in HTC, the Cx43 is visualized only on cytoplasm. In Orthogonal sections of HTC-Cx43 (D), the strong Cx43 immunoreaction in mitotic cell surface shows hemichannels formation. The nuclei were stained with Propidium Iodide (red).

The distribution of Cx43 was determined in confluent cultures submitted to immunofluorescence reactions. In BRL3A cells the connexin 43 was localized in the cytoplasm and also at the cell surface, forming gap junction structures in cell-to-cell contact areas (Figure 4A). HTC cells showed a diffuse Cx43 pattern in the cytoplasm. There was no evidence of gap junction structures at the plasma membrane (Figure 4B). Interestingly, transfected cells (HTC-Cx43) presented a lower Cx43 cytoplasm staining than the HTC cells, however Cx43 was observed at the plasma membrane in cell-to-cell contact areas (Figure 4C and 4D).

**Figure 5:**
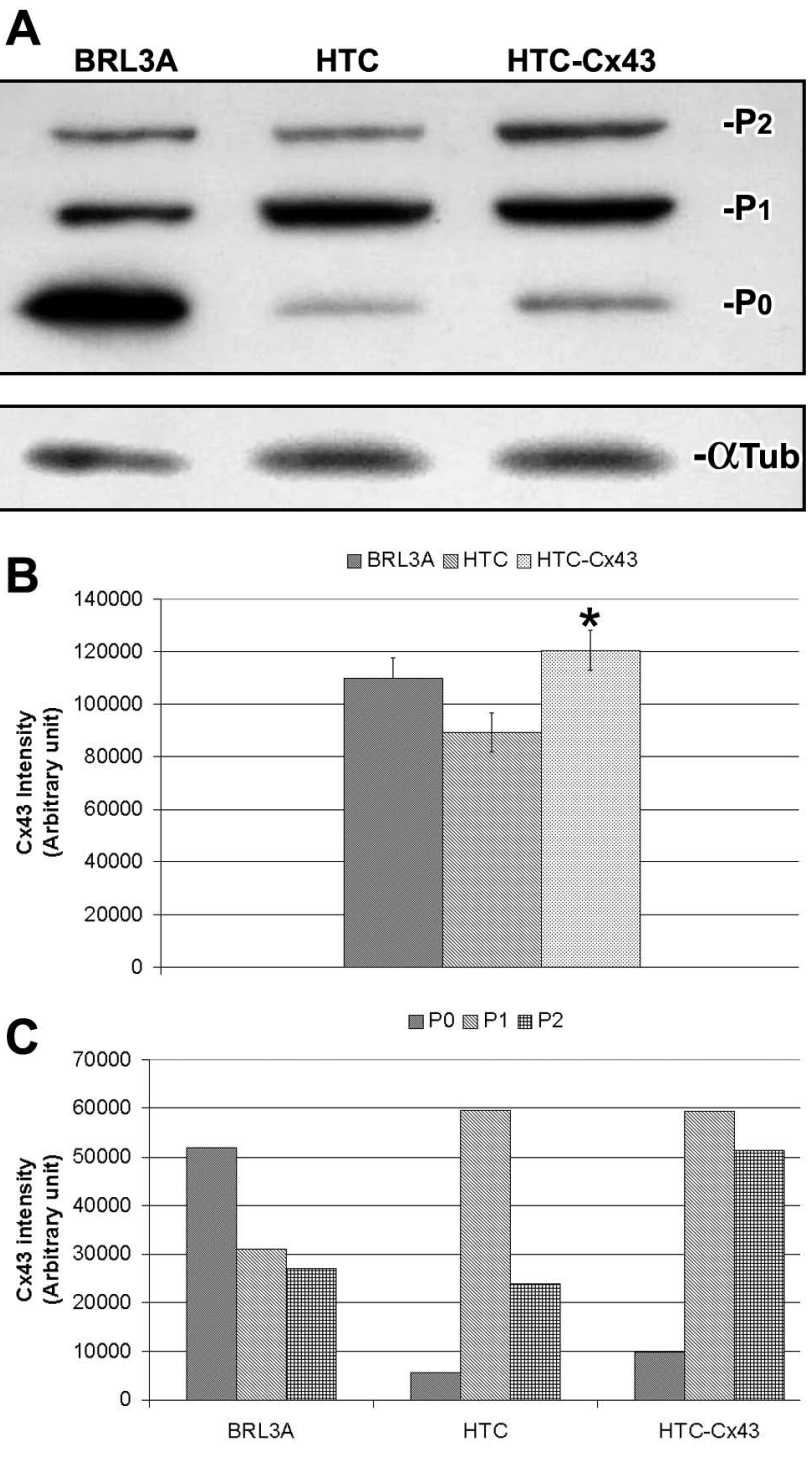
**Connexin43 expression**: (A) Western blotting of Cx43 and its respective densytometric analyses (B and C) showing total Cx43 expression increased in HTC-Cx43 when compared to HTC cells. Non-phosphorylated form (P0) was preferentially observed in BRL3A. While in HTC and HTC-Cx43 phosphorylated form (P1) was predominant. However in transfected cells (HTC-Cx43) there was increasing of the non-phosphorylated and phosphorylated forms (P0 and P2, respectively) when compared to HTC cells. (* p < 0.05).

The "western" blot analysis performed confirmed the expression of the connexin 43 protein in the BRL3A, HTC and HTC-Cx43 cells. Three immunoreactive bands were observed, which corresponded to nonphosphorylated (P0) and phosphorylated (P1 and P2) forms (Figure 5A). Total Cx43 expression in HTC-Cx43 was approximately 35% higher than HTC (Figure 5B). In the BRL3A cell line the P0 band presented the highest intensity. While in the HTC cell line, the P1 band was predominant. The expression profile of Cx43 in HTC-Cx43 cells was very interesting, since it presented similar intensity of the P1 band to HTC cells; however P0 and P2 bands were more intense in HTC-Cx43 cells than HTC cells (1.7 fold and 2.1 fold, respectively – see Figure 5C).

### Gap Junction Intercellular Communication Capacity

**Figure 6:**
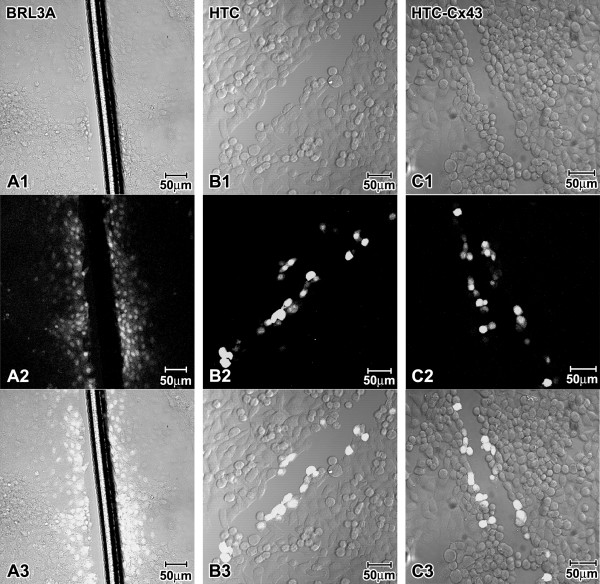
**Scrape loading/dye transfer assay performed in BRL3A, HTC and HTC-Cx43**. DIC images in A1, B1 and C1, fluorescence images in A2, B2 and C2, merge in A3, B3 and C3. BRL3A presented good communication capacity (at least 5 rows of fluorescent cells from scrape were observed (A). HTC and HTC-Cx43 were deficient in GJIC. Fluorescent cells were visualized only in areas near to scrape.

The functional gap junction assay was performed by Scrape Loading and Dye Transfer (SL/DT) in confluent cultures when the cell-to-cell contacts were well established. The BRL3A cell line showed good GJIC capacity. We observed at least 5 dye-coupled cell rows on either side of the scrape line in BRL3A cell line (Figure 6A), what shows the good ability of these cells to transfer the fluorescent dye to their neighboring cells. HTC and HTC-Cx43 cell lines were deficient in GJIC, considering that the fluorescent dye was only observed in cells near the scrape (Figure 6B and 6C). Thus, cell communication patterns observed in HTC and HTC-Cx43 were very similar suggesting that the exogenous Cx43 expression did not contribute to restore cell communication levels.

## Discussion

The connexin genes are considered to be tumor suppressors with two distinct forms of action: the first and more traditional idea is that GJs transmit important signal molecules that regulate cell proliferation, while the second and more recent view acknowledges the Cxs as proteins in their own right and mediate GJ-independent effects [18-20]. In this work we evaluated the effects of exogenous Cx43 expression on hepatocarcinoma cell proliferation and phenotype. Both the attainment of transfected cells and the inclusion of the normal cell line (BRL3A) which presents typical morphology of normal epithelial cells, good GJIC capacity via Cx43 and contact growth inhibition, was important in this study. The results showed that Cx43 cDNA transfected cells (HTC-Cx43) presented a lower proliferation rate than non-transfected cells (HTC). Our data are in accordance with those of other authors who observed a decrease in the cell proliferation rate in tumor cells after Cx43 transfection [11-13,20].

Recently Zhang et al. [21] showed that an increase in Cx32 expression inhibits the cell proliferation rate of human hepatoma cells (HuH7), contrary to Cx43 exogenous expression which contributed to increase the cell proliferation capacity. The authors did not investigate the cellular localization of the Cx43, an important aspect to be considered when proliferative behavior is evaluated. On the other hand, Li et al. [22] showed that the cytoplasm accumulation of Cx32 enhances motility and metastasis ability of HuH7 cells, despite the fact that Cx32 is considered to be a tumor suppressor. In the present work, we observed about 35% more Cx43 protein expression in HTC-Cx43 than HTC cells. Curiously, we also evidenced Cx43 protein on the cell surface in contrast to HTC cells that only presented Cx43 distributed on cytoplasm. Therefore, an increase in the protein levels was critical to Cx43 delivery to the plasma membrane in HTC-Cx43 cells.

Despite the fact that Cx43 has been observed in cell-to-cell contact areas in HTC-Cx43 cells, we did not evidence significant difference in the GJIC capacity when compared to HTC cells. These results suggest that the effect of the exogenous Cx43 expression on cell proliferation was GJIC-independent. Similar results were described by Huang et al.[20] who observed inhibition of the growth in human glioblastoma cell line after transfection with Cx43 cDNA without restoration of the GJIC.

Connexin 43 is a substrate for several kinases and can be phosphorylated at different sites [23]. Phosphorylation is implicated in the regulation at several stages of the Cx43 lifecycle (intracellular trafficking, connexon assembly and disassembly, insertion in the plasma membrane and degradation) [24-26]. Depending on the degree of phosphorylation, Cx43 exhibits different electrophoretic mobility in SDS-PAGE. Specific phosphorylation events induce conformational changes in phosphorylated forms of Cx43 which are critical to slower-migrating in SDS-PAGE [25]. We therefore investigated what Cx43 form is preferentially expressed in BRL3A since it presents normal transport of Cx43 to membrane. The predominant Cx43 form expressed in BRL3A was the nonpophosphorylated form (P0) in contrast to HTC cells which presented mainly the phosphorylated form (P1). Similar results were obtained by Udaka et al.[27] who found that the nonphosphorylated (P0) Cx43 form was predominantly expressed in normal lung in contrast to lung tumor. In the present work, HTC-Cx43 presented similar expression levels of the P1 form compared to HTC. However P0 and P2 forms were more expressed (1.7 fold and 2.1 fold, respectively). The changes observed in the phosphorylation pattern might have contributed to improve Cx43 oligomerization into connexon and its delivery to the plasma membrane.

Several reports have noticed that Cx43 can interact with several proteins including tight junction proteins, adherent junction proteins and cytoskeletal proteins [23]. Confocal microscope analyses evidenced reorganization of the microfilaments in HTC-Cx43 cells; actin filaments formed network and many stress fibers as well as cell flattening were observed. These changes were probably related to increased cell-to-cell and cell-to-substratum interaction. Similar features were observed when HTC cells were treated with *all-trans*-Retinoic Acid, an agent that induces cell differentiation (data not shown). Thus, we cannot exclude the possibility that the exogenous Cx43 stimulated the expression of molecules involved in the cell differentiation process and cell cycle control. Zhang et al. [28,29] demonstrated that G1/S cell cycle transition of osteosarcoma cells was inhibited after a Cx43 transfection as a consequence of the increase in p27 level and inhibition of S-phase kinase associated protein (SKp2). In the present work, the increase in G0/G1 cell populations observed in the HTC-Cx43 cells is in accordance with results presented by other authors. We addressed that growth control and phenotypic changes observed in rat hepatocarcinoma cells were mediated by exogenous Cx43 expression and that these phenomena were gap junction-independent.

## Conclusion

The data presented in this work demonstrate that the expression of exogenous Cx43 decreases the growth of rat hepatocellular carcinoma cells and contributes to reversion of the transformed phenotype. These effects were independent of the GJIC and were probably associated with phosphorylation pattern changes and redistribution of Cx43. The present data support the idea that Cx43 can act in growth suppression through an alternative mechanism that is independent of GJIC.

## Abbreviations

GJIC: gap junctional intercellular communication; Cx26: connexin 26; Cx32: connexin32; Cx43: connexin43; DMEM: Dulbecco's Modified Eagle's Minimum Essential Medium; FBS: Fetal Bovine Serum; BrdU: Bromodeoxyuridine; PBSA: phosphate-buffered saline; FITC: fluorescein isothiocyanate; TRITC: Tetramethylrhodamine-5-(and6)- isothiocyanate.

## Competing interests

The authors declare that they have no competing interests.

## Authors' contributions

MI: Cellular morphology and proliferative behavior characterization as well as connexin expression and GJIC and wrote the manuscript. RASF: Cx43 transfection and proliferative behavior of HTC-Cx43 cell line. SCP: Cx43 cDNA amplification and recombinant plasmid construction. GMMS: Experimental design, conception and manuscript preparation. All authors read and approved the final manuscript.
